# Non-neuromodulatory Optogenetic Tools in Zebrafish

**DOI:** 10.3389/fcell.2020.00418

**Published:** 2020-06-03

**Authors:** Adam Varady, Martin Distel

**Affiliations:** St. Anna Children's Cancer Research Institute, Innovative Cancer Models, Vienna, Austria

**Keywords:** zebrafish, optogenetics, non-neural optogenetics, synthetic biology, gene expression, protein localization, cell signaling

## Abstract

The zebrafish (*Danio rerio*) is a popular vertebrate model organism to investigate molecular mechanisms driving development and disease. Due to its transparency at embryonic and larval stages, investigations in the living organism are possible with subcellular resolution using intravital microscopy. The beneficial optical characteristics of zebrafish not only allow for passive observation, but also active manipulation of proteins and cells by light using optogenetic tools. Initially, photosensitive ion channels have been applied for neurobiological studies in zebrafish to dissect complex behaviors on a cellular level. More recently, exciting non-neural optogenetic tools have been established to control gene expression or protein localization and activity, allowing for unprecedented non-invasive and precise manipulation of various aspects of cellular physiology. Zebrafish will likely be a vertebrate model organism at the forefront of *in vivo* application of non-neural optogenetic tools and pioneering work has already been performed. In this review, we provide an overview of non-neuromodulatory optogenetic tools successfully applied in zebrafish to control gene expression, protein localization, cell signaling, migration and cell ablation.

## Introduction

Experimental control over protein function is an invaluable asset to dissect cellular processes on the molecular level. Conventional means of conditionally inducing protein activity, e.g., by small molecules or by heatshock, provide temporal control, but are typically limited in their spatial resolution. Optogenetic techniques emerged as a highly precise way to establish spatiotemporal control over protein activity by light.

**Figure 1:**
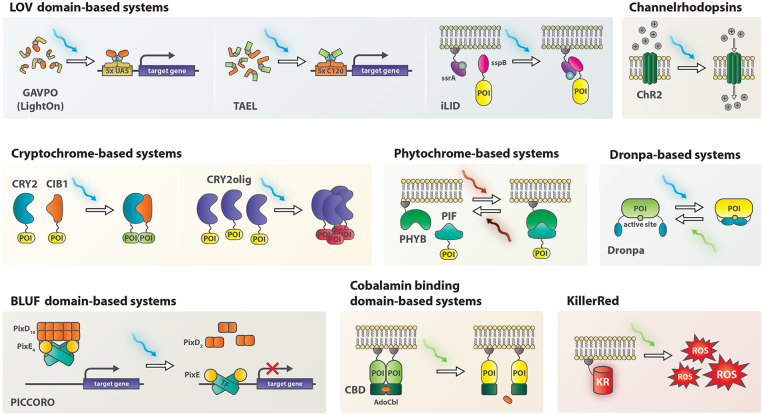
Schematic representation of non-neuromodulatory optogenetic systems applied in zebrafish. AdoCbl, 5′-deoxyadenosylcobalamin; CBD, cobalamin binding domain; ChR2, channelrhodopsin 2; CIB1, cryptochrome interacting bHLH 1; CRY2, cryptochrome 2; KR, KillerRed; PHYB, Phytochrome B; PIF, phytochrome interacting factor; POI, protein of interest; ROS, reactive oxygen species; TF, transcription factor.

Optogenetics was initially applied in neurobiology by ectopic expression of channelrhodopsins (ChRs), light-sensitive ion-channels, in neuronal cells (Boyden et al., 2005). ChRs depolarize neurons upon illumination and thereby modulate neuronal activity. Since then, the field has evolved to include non-neural applications. At the core of the new toolkit are light-sensitive proteins or protein-domains of bacterial, fungal and plant origin, such as phytochromes (e.g., PHYB-PIF), blue light using flavin (BLUF) domain proteins, cryptochromes (e.g., CRY2-CIB1) and light oxygen voltage (LOV) domains (Figure 1) (Ni et al., 1999; Harper et al., 2003; Liu et al., 2008; Yuan and Bauer, 2008). Irradiation with light of an appropriate wavelength causes conformational changes, commonly resulting in dimerization or oligomerization of the proteins. Careful engineering of photosensitive domains into enzymes, transcription factors or other proteins of interest endowed light-mediated control over the conformation of these proteins and resulted in light-activatable genetic tools for a variety of applications such as control over gene expression, genome editing, and protein relocalization (Beyer et al., 2015; Buckley et al., 2016; Polesskaya et al., 2018).

Zebrafish are an excellent model for optogenetic *in vivo* applications, since they are transparent at embryonic and larval stages and develop extra-uterine, providing light accessibility to the entire organism (Simmich et al., 2012). This optical clarity has been exploited to directly monitor fluorescently tagged cells and proteins in the living organism, hereby gaining great insights into cellular and subcellular processes during development and disease. In addition to this passive observation, novel optogenetic tools now enable manipulation of biological processes *in vivo* with the possibility of a direct readout of the effects. In this review, we summarize non-neuromodulatory optogenetic tools, which have recently been applied in zebrafish to control gene expression, cell migration, protein localization, signaling pathway activity and cell death (Table 1). Many of them can be readily combined with existing genetic systems like Gal4/UAS and established transgenic strains, making them a powerful addition to the genetic toolbox in zebrafish.

**Table 1:** Overview of non-neural optogenetic tools used in zebrafish.

**Protein/dimer system**	**Co-factors/chromophores**	**Reported activation wavelength (nm)**	**Binding/activation time**	**Unbinding/deactivation time**	**Mechanism of action**	**Applications in Zebrafish**	**References**
CRY2-CIB1	FAD	450-488	<1 s	12 min	Light-induced heterodimer (or CRY2 homo-oligomer), disruption in dark-state	Gal4-UAS-mediated gene expression in luciferase reporter assay	Liu et al., 2008, 2012
TAEL, EL222	FMN	460-470	<10 s (EL222)	<50 s (EL222)	Light-induced homodimer, disruption in dark-state	C120-promoter-mediated gene expression; induction of Cas9 expression for mosaic gene knockout	Motta-Mena et al., 2014; Reade et al., 2017
LightOn (GAVPO)	FAD, FMN	450-470 (<500)	Seconds^**^	2 h	Light-induced homodimer, disruption in dark-state	UAS-promoter-mediated gene expression for inducible cell ablation systems	Schwerdtfeger and Linden, 2003; Wang et al., 2012; Mruk et al., 2019
PICCORO (PixD/PixE)	FAD, FMN	472^*^ (320–500)	Seconds^**^	Minutes^**^	Dark-state heterooligomerization, light-induced oligomer-dissociation into homodimers	Light-mediated control over the activity of a dominant negative transcription factor	Yuan and Bauer, 2008; Masuda et al., 2013; Masuda and Tanaka, 2016
PA-Rac (LOV)	FMN	458–473^*^	Seconds^**^	Tens of seconds^**^	Light-induced Ras-GTPase-activity	Control over neutrophil movement	Wu et al., 2009; Yoo et al., 2010
ChR2	all-trans-Retinal	450–490	-	-	Cation-channel, modulation of cellular ion concentrations	Perturbation of pigment migration	Boyden et al., 2005; Aramaki and Kondo, 2019
PHYB-PIF	PCB	630-664	6.5 s	> 2 h (dark); 46.9 s (> 740 nm)	Light-induced heterodimer, stable in dark-state, disruption upon >740 nm light illumination	Gene expression in a luciferase assay, subcellular protein relocalization	Ni et al., 1999; Beyer et al., 2015; Buckley et al., 2016
CRY2olig	FAD	473^*^	15-75 s (t 1/2)	23 min (t 1/2)	Light-induced homooligomerization	Localized clustering of TDP-43 protein in neuronal cells	Bugaj et al., 2013; Lee et al., 2014; Asakawa et al., 2019
iLID	FMN	488^*^	<1 min	<2 min	Light-induced heterodimer, disruption in dark-state	Induction of mitophagy by protein relocalization	Guntas et al., 2015; D'Acunzo et al., 2019
Opto-Fz7	Rhodopsin	488^*^	N.A.	N.A.	Light-induced Fz7 activity	Direction of cell migration during gastrulation	Capek et al., 2019
Opto-Acvr1b/2b (LOV)	FMN	458^*^	Seconds^**^	Minutes^**^	Light-induced dimerization, leading to smad 2/3 phosphorylation and target gene expression	Control over Nodal signaling in zebrafish embryos during gastrulation	Sako et al., 2016
CBD	AdoCbl	540-550	N.A.	N.A.	Dark-state assembled heterodimer, disruption upon activating illumination	Disruption of constitutively active mFGFR1	Jost et al., 2015; Kainrath et al., 2017
psMEK (Dronpa)	-	400	Seonds^**^	500 nm; Seconds^**^	Homodimerization upon 400 nm illumination, dissociation upon 500 nm illumination	Modulation of MEK activity	Zhou et al., 2017; Patel et al., 2019
KillerRed	-	520–590	-	-	Photosensitizer, chromophore generating high amounts of ROS upon activating illumination	Directed cell ablation in heart, kidney and spinal cord	Bulina et al., 2006; Teh et al., 2010; Buckley et al., 2017; Formella et al., 2018

*AdoCbl, 5′-deoxyadenosylcobalamin; CBD, cobalamin binding domain; ChR, Channel rhodopsin; CIB1, cryptochrome interacting bHLH 1; CRY2, cryptochrome 2; FAD, flavin adenine dinucleotide; FMN, flavin mononucleotide; Fz7, frizzled 7; iLID, improved light-inducible dimer; LOV, light oxygen voltage; PA-Rac, photo-activatable Rac; PCB, phycocyanobilin; PhyB, phytochrome B; PICCORO, PixD complex dependent control of transcription; PIF, phytochrome interacting factor; ROS, reactive oxygen species; TAEL, TA4-EL222; TDP-43, transactivation response element DNA-binding protein 43*.

* *Single wavelength used in experiments, full range not tested in cited literature*.

** *Approximate activation times taken from Optobase (https://www.optobase.org/switches/) Kolar et al., 2018*.

## Gene Expression

Control over gene expression is beneficial to interrogate gene function and to model diseases including neurodegeneration and cancer. Temporal control is typically achieved in zebrafish by using either compound-activated systems like tamoxifen for estrogen receptor fusions or heatshock promoters (Mayrhofer and Mione, 2016). Optogenetic gene expression systems additionally offer spatial control and here we present their current applications in zebrafish.

### Cryptochrome 2 (CRY2-CIB1)

The *Arabidopsis thaliana* photoreceptor protein cryptochrome 2 (CRY2) heterodimerizes with the CRY-interacting basic-helix-loop-helix (bHLH) transcription factor 1 (CIB1) upon blue light illumination (Liu et al., 2008). This heterodimerization was exploited to create a light-inducible Gal4 system by fusing the Gal4 DNA binding domain to CRY2 and the Gal4 activation domain containing VP16 to CIB1. A luciferase-based readout revealed that blue-light illumination for 2 h, but not red light, induced luciferase expression in zebrafish with this system (Liu et al., 2012).

### LOV Domains

#### EL222/C120 and TAEL

EL222 is a LOV domain-containing light-inducible transcription factor from *Erythrobacter litoralis*. Blue light induces homodimerization of EL222 and subsequent binding to its regulatory element C120 activates transcription of downstream genes. EL222 was adapted for eukaryotic use by adding a nuclear localization sequence and the *herpes simplex* virus-derived VP16 transcriptional activation domain resulting in VP-EL222 (Motta-Mena et al., 2014). Injection of VP-EL222 mRNA together with a C120:mCherry reporter into zebrafish demonstrated that blue light effectively mediates gene expression in this system. As some toxicity was observed with VP-EL222 it was further optimized for applications in zebrafish by exchanging the VP16 transcriptional activation domain with TA4, which is better tolerated in zebrafish, resulting in TA4-EL222 (TAEL) (Distel et al., 2009; Reade et al., 2017).

The TAEL system was used to control the expression of several genes, including *sox32* to convert ectoderm to endoderm, *lefty* to modulate Nodal signaling and Cas9 for light-induced mosaic gene knock out. Importantly, selective illumination using either an epifluorescene, a confocal or light sheet microscope or a digital mirror device (DMD) demonstrated that TAEL endows temporal and spatial control over gene expression in zebrafish (Reade et al., 2017).

#### LightOn/GAVPO

The LightOn gene expression system is based on a synthetic protein termed GAVPO (Wang et al., 2012). GAVPO contains a Gal4 DNA binding domain, a p65 transactivation domain and the small fungal protein Vivid including its LOV domain for light-mediated dimerization (Schwerdtfeger and Linden, 2003). As Gal4 binds to UAS sites as a homodimer, its transcriptional activity can be controlled by light using the LightOn system (Wang et al., 2012). Illumination with blue light triggers homodimerization of GAVPO. The dimer then binds to UAS sites to induce expression of a gene of interest. The LightOn system was applied in zebrafish to gain spatiotemporal control over expression of two cell ablation systems, nitroreductase and the cytotoxic viral ion channel M2H37A (Mruk et al., 2019). GAVPO-mediated nitroreductase expression induced apoptosis in embryos when treated with metronidazole and irradiated with blue light. In addition, blue-light induced GAVPO-triggered expression of M2H37A leading to necrotic cell death and developmental defects in zebrafish embryos, which could be rescued by addition of the antiviral channel blocker rimantadine. Furthermore, a transgenic GAVPO strain (*elavl3:GAVPO*) was successfully established, demonstrating that GAVPO expression can be tolerated in zebrafish, although a previous study reported toxicity of GAVPO (Reade et al., 2017).

### PICCORO

The BLUF domain-based “PixD complex dependent control of transcription” (PICCORO) system was engineered to directly control transcription factor activity by protein localization (Masuda et al., 2013). PixD is a bacterial blue-light photoreceptor that interacts with the response regulator-like protein PixE and forms PixD_10_-PixE_4_ oligomers in the dark (Masuda and Tanaka, 2016). Illumination with blue light dissociates the large oligomeric complexes into PixD dimers and PixE monomers, triggered by changes in protein conformation (Yuan and Bauer, 2008) (Figure 1).

The PICCORO system was applied in zebrafish for light-mediated control of an engineered transcriptionally repressive version of the transcription factor No Tail (Ntl), which is important for tail tissue formation (Masuda et al., 2013). For this, the DNA binding domain of Ntl and the repressor domain of Engrailed were fused, resulting in the transcriptional repressor Ntl-EnR. When expressed in zebrafish, Ntl-EnR led to the typical “ntl” phenotype with missing tail tissues. To achieve light-dependent transcriptional repression, the N-terminus of PixE was fused to Ntl-EnR and the construct was named NtlPixE. When NtlPixE was expressed in a transgenic strain also ubiquitously expressing PixD (tg(EF1α:PixD)), transcriptional repression and the resulting “ntl” phenotype was ameliorated in the dark, as PixD_10_-NtlPixE_4_ oligomers would form and sequester NtlPixE away from its DNA binding site. Blue light illumination could restore the “ntl” phenotype, revealing direct light-mediated control of a chimeric transcription factor in zebrafish.

## Cell Migration

The cytoskeleton is a major contributor to cell migration and morphology changes. Several optogenetic tools have been established to control cytoskeleton dynamics in a precise manner, offering possibilities to investigate the mechanisms underlying migration.

### Photoactivatable Rac

In an effort to investigate phosphoinositide 3-kinase (PI3K)-dependent movement of neutrophils in zebrafish, photo-activatable Rac (PA-Rac) was employed (Yoo et al., 2010). PA-Rac was engineered by fusion of a phototropin-derived LOV domain to Rac1 to sterically inhibit Rac function. Illumination with blue-light restores Rac activity and induces directed cell movement *in vitro* (Wu et al., 2009). In zebrafish embryos expressing PA-Rac in neutrophils, precise regulation of directional neutrophil migration was demonstrated using laser illumination. By this means, neutrophils could even be diverted away from wounds, opening up new possibilities to investigate cellular functions in inflammation (Yoo et al., 2010).

### Channelrhodopsin (ChR)

In addition to controlling neuronal activity, ChRs were also applied in modulating cell migration. Through tissue-specific expression of ChR2, a blue-light-reactive cation channel protein, zebrafish pigmentation patterns were temporarily disarranged by illumination due to induced migration of pigment melanophores (Aramaki and Kondo, 2019).

## Protein Localization

Tight regulation of protein localization in cellular compartments is crucial for many biological processes, and its dysregulation can lead to disease. Studying these processes by manipulation of protein localization requires tools, which act rapidly and with high spatial resolution. Several optogenetic systems have been developed to shuttle proteins between specific subcellular compartments or to form clusters among each other.

### Phytochrome B (PHYB-PIF)

The photoreactive *Arabidopsis* proteins phytochrome B (PHYB) and the bHLH transcription factor phytochrome interaction partner (PIF) heterodimerize upon red light illumination in the presence of the chromophore phycocyanobilin (PCB) (Ni et al., 1999). The interaction remains stable in the dark for hours after activation. Furthermore, illumination with far-red light dissociates the dimers.

In an elegant study, PHYB-PIF was applied to control nuclear localization of a synthetic transcription factor. Here, PHYB was fused to a VP16 activation domain and a heterodimerizing antiparallel leucine zipper. The tetracycline repressor (TetR) DNA binding protein was attached to PHYB-VP16 via a corresponding leucine zipper. PIF3, which possesses a nuclear localization signal, was used to translocate this chimeric transcription factor into the nucleus in zebrafish upon red-light illumination, hereby controlling the expression of luciferase. PCB was added to the medium in this study (Beyer et al., 2015). The PHYB-PIF system was further optimized for application in zebrafish by truncation of PHYB for efficient expression and injection of PCB to deliver the chromophore into cells beyond the first layer of tissue (Buckley et al., 2016). After these adjustments, PHYB was linked to CAAX for cell-membrane binding, while PIF6 was fused to enhanced green fluorescent protein (EGFP) or Pard3 (Buckley et al., 2016). Red light illumination of a region of interest triggered protein shuttling of PIF6-EGFP or PIF6-Pard3 to the cell membrane, respectively. Activation and binding of the association partners were reported to occur in a matter of seconds, with unbinding kinetics of less than a minute after far-red light illumination.

### CRY2olig

CRY2 self-clusters when illuminated, which is an undesirable feature for its function in the CRY2/CIB1 heterodimer but was found useful to induce protein relocalization, leading to the development of the CRY2olig protein clustering system (Bugaj et al., 2013; Lee et al., 2014). Functionality of this system in zebrafish was demonstrated by expression of mRFP1-tagged CRY2olig, which clustered *in vivo* upon blue-light stimulation (Asakawa et al., 2019). To study the effects of transactivation response element DNA-binding protein 43 (TDP-43) oligomerization in amyotrophic lateral sclerosis in zebrafish, the zebrafish *tardp* gene was inserted into the mRFP1-CRY2olig construct and named opTDP-43z. Physiological function of opTDP-43z was unaffected by the CRY2olig-fusion compared to wildtype *tardp*. In dark-state, opTDP-43z was detected mainly in cell nuclei, but started relocalizing to and aggregating in the cytosol after a blue-light stimulus. Light-induced clustering of opTDP-43z expressed in neurons perturbed axonal outgrowth, increased myofiber denervation frequency and seeded non-optogenetically induced delocalization of TDP-43 (Asakawa et al., 2019).

CRY2olig promises to be a general tool to investigate protein-clustering related diseases or to control protein activity by delocalization.

### Improved Light-Inducible Dimer (iLID)

The improved light-inducible dimer (iLID) system consisting of the *E.coli* peptides SspB and SsrA fused to the *Avena sativa* LOV2 domain was engineered for a high change in affinity for their respective binding partner upon blue light stimulation (Guntas et al., 2015). The iLID system offers rapid activation and deactivation kinetics and lacks homo-monomerization. iLID was applied in zebrafish to regulate mitophagy (D'Acunzo et al., 2019). To achieve this, SsrA was fused to Venus and actin assembly inducing protein (ActA). SspB was fused to RFP and autophagy and beclin-1 regulator 1 (AMBRA1). Venus-ssrA-ActA was tethered to mitochondrial outer membranes while AMBRA1-RFP-sspB was present in the cytosol in the dark. Blue-light illumination triggered association of the two proteins, resulting in relocalization of AMBRA1 to the mitochondrial membrane, inducing mitophagy and leading to a loss of mitochondrial mass (D'Acunzo et al., 2019).

## Controlling Cell Signaling and Protein Activity

Precise spatiotemporal control over signaling pathway activity allows for detailed investigation of their role in cellular processes. In contrast to studying the function of proteins and pathways by transient or permanent changes in gene expression, manipulation of protein activity results in an immediate effect. Light is an ideal activator for fast-acting interference in signaling and multiple optogenetic proteins have been engineered to modulate major pathways like Wnt, Nodal or FGF in zebrafish.

### Rhodopsin-Based Opto-Fz7 to Study Non-canonical Wnt-Fz/PCP Signaling

The non-canonical Wnt receptor frizzled 7 (Fz7) was engineered to be activated by light independent of ligand binding. To achieve this, the intracellular loop 3 and the C-terminus of the light sensitive receptor rhodopsin were replaced by the corresponding intracellular domains of Fz7 to create Opto-Fz7 (Capek et al., 2019). Applying Opto-Fz7 revealed that non-canonical Wnt-Frizzled(Fz)/planar cell polarity (PCP) signaling provides a permissive signal for directed migration of mesenchymal cells during zebrafish gastrulation.

### LOV-Domain-Mediated Optogenetic Control Over Nodal Signaling

In order to control Nodal signaling and study its temporal role in mesendoderm induction in zebrafish, light-sensitive Nodal receptors were engineered by fusing the LOV domain of aureochrome 1 from *Vaucheria frigida* with the C-terminal intracellular domains of Nodal receptors Acvr1b and Acvr2b (Sako et al., 2016). Blue light controlled the dimerization of Opto-Acvr1b and 2b, upon which Nodal signaling was activated resulting in phosphorylation of Smad2 and expression of downstream target genes like goosecoid (Sako et al., 2016).

### Cobalamin Binding Domains (CBDs) to Interrogate FGF Signaling

In contrast to the LOV domain based Nodal receptors Opto-Acvr1b and 2b, which dimerize upon blue light illumination, a green light-mediated de-dimerization system was used to inactivate FGF signaling. This system is based on cobalamin binding domains (CBDs) of bacterial CarH transcription factors, which are present as dimers in the dark but dissociate upon illumination with green light (Jost et al., 2015). For CBD-dimer assembly in the dark, 5′-deoxyadenosylcobalamin (AdoCbl) is required, which is cleaved from the protein upon green light illumination. A constitutively active FGF receptor, which can be inactivated by green light, was created by fusing murine mFGFR1 to the CBD from *M. xanthus* (MxCBD) (Kainrath et al., 2017). Injection of mFGFR1-MxCBD into zebrafish embryos together with AdoCbl led to severe malformations when kept in the dark consistent with hyperactive FGF signaling. However, green light illumination after injection of mFGFR1-MxCBD and AdoCbl completely rescued the phenotype, demonstrating that excessive FGF signaling could be inactivated *in vivo* (Kainrath et al., 2017).

### DRONPA-Caged MEK1 to Control ERK Activity

In order to render MEK1 light-inducible, photo-dimerizable Dronpa (pdDronpa) was used to cage MEK1's active site in a single chain construct termed psMEK1tight (Zhou et al., 2017). Upon illumination with 500 nm Dronpa de-dimerizes and allows psMEK1 to bind ERK, whereas 400 nm light re-establishes the caged conformation. When applied in zebrafish psMEK1tight showed surprisingly no effect during early development (Patel et al., 2019). However, once optimized by introduction of an activating mutation found in cancer (E203K), a non-leaky light-controlled MEK1 (psMEK^E203K^) for *in vivo* investigation of MAPK signaling in zebrafish could be generated.

## Optical Cell Ablation Systems

Methods to ablate distinct cells are beneficial to study cell function or regeneration. Current chemical/ genetically encoded systems are limited in their spatial resolution, but optogenetic tools offer unique selectivity of target cells.

### KillerRed

KillerRed (KR) is a fluorescent protein, which produces large doses of reactive oxygen species (ROS) upon green light illumination, leading to apoptosis (Bulina et al., 2006). KR has been implemented in zebrafish for ROS-induced cell ablation *in vivo*. Transgenic fish lines with membrane-tethered KR expressed either in the heart or kidney were generated (Teh et al., 2010; Buckley et al., 2017). ROS levels could be controlled to inflict damage to cell membranes or cause death of illuminated KR-positive cells. Additionally, KR-activation in the heart closely resembled heart failure in humans (Teh et al., 2010). For studying neurodegeneration, a transgenic zebrafish model expressing KR in spinal cord neurons was generated, providing single cell control over cell ablation (Formella et al., 2018).

The possibility to control area, intensity and duration of ROS expression makes KR transgenic models a useful tool, not only for directed cell ablation, but also to study effects of free radicals on different tissues.

## Conclusion and Perspectives

Applying optogenetics in zebrafish offers unprecedented spatiotemporal control over protein function in a living vertebrate model organism. We have summarized recent studies using chimeric proteins engineered to endow light-mediated control over their activity in biological processes ranging from gene expression, cell migration, mitophagy and signaling pathways to cell ablation.

So far, LOV and BLUF domain-, pdDronpa-, CRY2-CIB-, PHYB-PIF-, and CBD-based systems have been successfully applied in zebrafish, revealing that zebrafish are generally permissive for various optogenetic systems (Table 1). Some of the systems needed zebrafish-specific optimization to ensure proper expression (e.g., truncation of the PHYB-PIF system Buckley et al., 2016) or to reduce toxicity (e.g., replacement of the transactivation domain of VP-EL222 to generate TAEL Reade et al., 2017).

LOV and BLUF domain-based systems as well as CRY-CIB are activated by blue light, CBD by green light and PHYB-PIF by red light, offering potential orthogonal applications, which have not been realized in zebrafish to date.

An important aspect to be considered is the availability of the co-factor for the respective optogenetic system in zebrafish. Blue-light activated systems typically rely on Flavin mononucleotides (FMN) or Flavin adenine dinucleotides (FAD), which are readily available in zebrafish cells (Table 1). However, cobalamins needed for CBD or PCB for PHYB-PIF have to be supplemented and as absorption from the medium is limited, these cofactors are ideally injected. Injection at the one cell-stage will result in dilution of the cofactor while cells are dividing and will thus limit the application of the system to early developmental stages. Possible ways to apply these systems at later stages are to engineer transgenic zebrafish strains, expressing the enzymes needed to produce the cofactor (e.g., mitochondria-localized cyanobacterial heme oxygenase 1 and PCB:ferredoxin oxidoreductase for PCB). This would greatly enhance the applicability of the PHYB-PIF system with its unique and elegant far-red light off-switch.

Some of the systems like LightOn (GAVPO) can be combined with available UAS strains for light-mediated control of a plethora of transgenes promising widespread use. The ideal optogenetic system for zebrafish still needs to be determined and further rounds of optimization of expression levels, toxicity and increased activity change will likely be required for their easy application. Nevertheless, the possibility to manipulate a biological process and to image the effects using real time reporters as readout, puts zebrafish in a prime position to take full advantage of the optogenetic toolkit and shine light on many biological processes.

## Author Contributions

AV and MD wrote the manuscript.

## Conflict of Interest

The authors declare that the research was conducted in the absence of any commercial or financial relationships that could be construed as a potential conflict of interest.

## Abbreviations

ActA: actin assembly inducing protein
AdoCbl: 5′-deoxyadenosylcobalamin
AMBRA1: autophagy and beclin-1 regulator 1
BLUF: blue light using flavin
bHLH: basic-helix-loop-helix transcription factor
CBD: cobalamin binding domain
ChR: Channel rhodopsin
CIB1: cryptochrome interacting bHLH 1
CRY2: cryptochrome 2
DMD: digital mirror device
FAD: flavin adenine dinucleotide
FMN: flavin mononucleotide
Fz(7): frizzled (7)
iLID: improved light-inducible dimer
KR: KillerRed
LOV: light oxygen voltage
MxCBD: *M. xanthus* CBD
Ntl: No Tail
PA-Rac: photo-activatable Rac
PCB: phycocyanobilin
PCP: planar cell polarity
PHYB: phytochrome B
PI3K: phosphoinositide 3-kinase
PICCORO: PixD complex dependent control of transcription
PIF: phytochrome interacting factor
RFP: red fluorescent protein
ROS: reactive oxygen species
TAEL: TA4-EL222
TDP-43: transactivation response element DNA-binding protein 43
TetR: tetracycline repressor
UAS: upstream activating sequence.

## References

[B1] Aramaki T. Kondo S. (2019). Method for disarranging the pigment pattern of zebrafish by optogenetics. Dev. Biol. 460, 12–19. 10.1016/j.ydbio.2018.12.019 30578760

[B2] Asakawa K. Handa H. Kawakami K. (2019). Optogenetic modulation of TDP-43 oligomerization fast-forwards ALS-related pathologies in the spinal motor neurons. Nat Commun. 11:1004. 10.1101/789057 32081878 PMC7035286

[B3] Beyer H. M. Juillot S. Herbst K. Samodelov S. L. Müller K. Schamel W. W.. (2015). Red light-regulated reversible nuclear localization of proteins in mammalian cells and zebrafish. ACS Synth. Biol. 4, 951–958. 10.1021/acssynbio.5b00004 25803699

[B4] Boyden E. S. Zhang F. Bamberg E. Nagel G. Deisseroth K. (2005). Millisecond-timescale, genetically targeted optical control of neural activity. Nat. Neurosci. 8, 1263–1268. 10.1038/nn1525 16116447

[B5] Buckley C. Carvalho M. T. Young L. K. Rider S. A. McFadden C. Berlage C.. (2017). Precise spatio-temporal control of rapid optogenetic cell ablation with mem-killerred in zebrafish. Sci. Rep. 7:5096. 10.1038/s41598-017-05028-2 28698677 PMC5506062

[B6] Buckley C. E. Moore R. E. Reade A. Goldberg A. R. Weiner O. D. Clarke J. D. W. (2016). Reversible optogenetic control of subcellular protein localization in a live vertebrate embryo. Dev. Cell 36, 117–126. 10.1016/j.devcel.2015.12.011 26766447 PMC4712025

[B7] Bugaj L. J. Choksi A. T. Mesuda C. K. Kane R. S. Schaffer D. V. (2013). Optogenetic protein clustering and signaling activation in mammalian cells. Nat. Methods 10, 249–252. 10.1038/nmeth.2360 23377377

[B8] Bulina M. E. Chudakov D. M. Britanova O. V. Yanushevich Y. G. Staroverov D. B. Chepurnykh T. V.. (2006). A genetically encoded photosensitizer. Nat. Biotechnol. 24, 95–99. 10.1038/nbt1175 16369538

[B9] Capek D. Smutny M. Tichy A. M. Morri M. Janovjak H. Heisenberg C. P. (2019). Light-activated frizzled7 reveals a permissive role of non-canonical wnt signaling in mesendoderm cell migration. Elife 8:e42093. 10.7554/eLife.42093 30648973 PMC6365057

[B10] D'Acunzo P. Strappazzon F. Caruana I. Meneghetti G. Di Rita A. Simula L.. (2019). Reversible induction of mitophagy by an optogenetic bimodular system. Nat. Commun. 10:1533. 10.1038/s41467-019-09487-1 30948710 PMC6449392

[B11] Distel M. Wullimann M. F. Köster R. W. (2009). Optimized Gal4 genetics for permanent gene expression mapping in zebrafish. Proc. Natl. Acad. Sci. U.S.A. 106, 13365–13370. 10.1073/pnas.0903060106 19628697 PMC2726396

[B12] Formella I. Svahn A. J. Radford R. A. W. Don E. K. Cole N. J. Hogan A.. (2018). Real-time visualization of oxidative stress-mediated neurodegeneration of individual spinal motor neurons *in vivo*. Redox Biol. 19, 226–234. 10.1016/j.redox.2018.08.011 30193184 PMC6126400

[B13] Guntas G. Hallett R. A. Zimmerman S. P. Williams T. Yumerefendi H. Bear J. E.. (2015). Engineering an improved light-induced dimer (ILID) for controlling the localization and activity of signaling proteins. Proc. Natl. Acad. Sci. U.S.A. 112, 112–117. 10.1073/pnas.1417910112 25535392 PMC4291625

[B14] Harper S. M. Neil L. C. Gardner K. H. (2003). Structural basis of a phototropin light switch. Science 301, 1541–1544. 10.1126/science.1086810 12970567

[B15] Jost M. Fernández-Zapata J. Polanco M. C. Ortiz-Guerrero J. M. Chen P. Y. Kang G.. (2015). Structural basis for gene regulation by a B12-dependent photoreceptor. Nature 526, 536–541. 10.1038/nature14950 26416754 PMC4634937

[B16] Kainrath S. Stadler M. Reichhart E. Distel M. Janovjak H. (2017). Green-light-induced inactivation of receptor signaling using cobalamin-binding domains. Angew. Chem. Int. Ed. 56, 4608–4611. 10.1002/anie.201611998 28319307 PMC5396336

[B17] Kolar K. Knobloch C. Stork H. Žnidarič M. Weber W. (2018). OptoBase: a web platform for molecular optogenetics. ACS Synth. Biol. 7, 1825–1828. 10.1021/acssynbio.8b00120 29913065

[B18] Lee S. Park H. Kyung T. Kim N. Y. Kim S. Kim J.. (2014). Reversible protein inactivation by optogenetic trapping in cells. Nat. Methods 11, 633–636. 10.1038/nmeth.2940 24793453

[B19] Liu H. Gomez G. Lin S. Lin S. Lin C. (2012). Optogenetic control of transcription in zebrafish. PLoS ONE 7:e50738. 10.1371/journal.pone.0050738 23226369 PMC3511356

[B20] Liu H. Yu X. Li K. Klejnot J. Yang H. Lisiero D.. (2008). Photoexcited CRY2 interacts with CIB1 to regulate transcription and floral initiation in arabidopsis. Science 322, 1535–1539. 10.1126/science.1163927 18988809

[B21] Masuda S. Nakatani Y. Ren S. Tanaka M. (2013). Blue light-mediated manipulation of transcription factor activity *in vivo*. ACS Chem. Biol. 8, 2649–2653. 10.1021/cb400174d 24063403

[B22] Masuda S. Tanaka M. (2016). PICCORO: a technique for manipulating the activity of transcription factors with blue light. Methods Cell Biol. 135, 289–295. 10.1016/bs.mcb.2016.03.009 27443931

[B23] Mayrhofer M. Mione M. (2016). The toolbox for conditional zebrafish cancer models. Adv. Exp. Med. Biol. 916, 21–59. 10.1007/978-3-319-30654-4_2 27165348

[B24] Motta-Mena L. B. Reade A. Mallory M. J. Glantz S. Weiner O. D. Lynch K. W.. (2014). An optogenetic gene expression system with rapid activation and deactivation kinetics. Nat. Chem. Biol. 10, 196–202. 10.1038/nchembio.1430 24413462 PMC3944926

[B25] Mruk K. Ciepla P. Piza P. A. Alnaqib M. Chen J. K. (2019). Targeted cell ablation in zebrafish using optogenetic transcriptional control. bioRxiv [Preprint]. 10.1101/730507 32414936 PMC7328002

[B26] Ni M. Tepperman J. M. Quail P. H. (1999). Binding of phytochrome B to its nuclear signalling partner PIF3 is reversibly induced by light. Nature 400, 781–784. 10.1038/23500 10466729

[B27] Patel A. L. Yeung E. McGuire S. E. Wu A. Y. Toettcher J. E. Burdine R. D.. (2019). Optimizing photoswitchable MEK. Proc. Natl. Acad. Sci. U.S.A. 116, 25756–25763. 10.1073/pnas.1912320116 31796593 PMC6926043

[B28] Polesskaya O. Baranova A. Bui S. Kondratev N. Kananykhina E. Nazarenko O.. (2018). Optogenetic regulation of transcription. BMC Neurosci. 19(Suppl. 1):12. 10.1186/s12868-018-0411-6 29745855 PMC5998900

[B29] Reade A. Motta-Mena L. B. Gardner K. H. Stainier D. Y. Weiner O. D. Woo S. (2017). TAEL: a zebrafish-optimized optogenetic gene expression system with fine spatial and temporal control. Development 144, 345–355. 10.1242/dev.139238 27993986 PMC5394756

[B30] Sako K. Pradhan S. J. Barone V. Inglés-Prieto Á. Müller P. Ruprecht V.. (2016). Optogenetic control of nodal signaling reveals a temporal pattern of nodal signaling regulating cell fate specification during gastrulation. Cell Rep. 16, 866–877. 10.1016/j.celrep.2016.06.036 27396324

[B31] Schwerdtfeger C. Linden H. (2003). VIVID is a flavoprotein and serves as a fungal blue light photoreceptor for photoadaptation. EMBO J. 22, 4846–4855. 10.1093/emboj/cdg451 12970196 PMC212719

[B32] Simmich J. Staykov E. Scott E. (2012). Zebrafish as an appealing model for optogenetic studies. Prog. Brain Res. 196, 145–162. 10.1016/B978-0-444-59426-6.00008-2 22341325

[B33] Teh C. Chudakov D. M. Poon K. L. Mamedov I. Z. Sek J. Y. Shidlovsky K.. (2010). Optogenetic *in vivo* cell manipulation in killerred-expressing zebrafish transgenics. BMC Dev. Biol. 10:110. 10.1186/1471-213X-10-110 21040591 PMC2989954

[B34] Wang X. Chen X. Yang Y. (2012). Spatiotemporal control of gene expression by a light-switchable transgene system. Nat. Methods 9, 266–269. 10.1038/nmeth.1892 22327833

[B35] Wu Y. I. Frey D. Lungu O. I. Jaehrig A. Schlichting I. Kuhlman B.. (2009). A genetically-encoded photoactivatable rac controls the motility of living cells. Nature 461, 104–108. 10.1038/nature08241 19693014 PMC2766670

[B36] Yoo S. K. Deng Q. Cavnar P. J. Wu Y. I. Hahn K. M. Huttenlocher A. (2010). Differential regulation of protrusion and polarity by PI(3)K during neutrophil motility in live zebrafish. Dev. Cell 18, 226–236. 10.1016/j.devcel.2009.11.015 20159593 PMC2824622

[B37] Yuan H. Bauer C. E. (2008). PixE promotes dark oligomerization of the BLUF photoreceptor PixD. Proc. Natl. Acad. Sci. U.S.A. 105, 11715–11719. 10.1073/pnas.0802149105 18695243 PMC2575306

[B38] Zhou X. X. Fan L. Z. Li P. Shen K. Lin M. Z. (2017). Optical control of cell signaling by single-chain photoswitchable kinases. Science 355, 836–842. 10.1126/science.aah3605 28232577 PMC5589340

